# Advances in the Diagnosis and Management of Congenital Heart Disease in Children

**DOI:** 10.3390/children10040753

**Published:** 2023-04-21

**Authors:** P. Syamasundar Rao

**Affiliations:** Children’s Heart Institute, University of Texas at Houston McGovern Medical School, Memorial Hermann Children’s Hospital, 6410 Fannin Street, Suite #425, Houston, TX 77030, USA; p.syamasundar.rao@uth.tmc.edu

The last five decades have witnessed an inordinate number of advances in the diagnosis and management of congenital heart defects (CHDs), as reviewed elsewhere [1]. These advances include the detection of CHDs using fetal echocardiography; the identification of critical CHD via the pulse oximetry screening of neonates before discharge from the hospital, in addition to conventional methods of CHD identification by routine history, physical examination, and simple laboratory studies, such as chest X-ray and electrocardiogram; the rapid transportation of the so-identified babies to tertiary care centers equipped to care for these vulnerable infants; accessibility to very sensitive noninvasive diagnostic techniques, namely, echocardiography and Doppler, three-dimensional (3D) echocardiography, magnetic resonance imaging (MRI), and computed tomography (CT); the availability and utilization of three-dimensional (3D) visualization technologies, including 3D printing, virtual reality, and augmented reality for surgical pre-planning; the introduction of percutaneous, catheter-based methodologies to address CHDs; developments in pediatric cardiac anesthesia for both percutaneous and surgical interventions; the enablement of intricate surgical techniques to provide corrective treatment to patients with complex CHDs with an alternative of successful palliation or heart transplantation; effective postoperative management; and diligent post-intervention follow-up. These advances have obtained positive results that improve the outcome of babies born with CHDs to the point that there are now more adult subjects with CHD than children. In this Special Issue on “Advances in the Diagnosis and Management of Congenital Heart Disease in Children”, some of these advances will be reviewed.

In the first paper, Vecchiato and associates from Padova and Naples, Italy, discuss the overshoot of the respiratory exchange ratio during recovery from maximal exercise testing in young patients with congenital heart disease (CHD) [2]. The authors state that overshoot of the respiratory exchange ratio (RER) following exercise is decreased in subjects with congestive heart failure (CHF). The objective of their investigation was to detect the occurrence of RER in children with CHD. This is a retrospective review of the results of cardiopulmonary exercise testing (CPET), with particular attention being paid to RER parameters. The RER at peak exercise, the highest RER amount achieved during recovery from exercise, the extent of the RER overshoot, and the linear slope of the RER increase at the conclusion of the CPET were evaluated. The study subjects included 93 children with CHD and 24 healthy children who were in age-matched controls with the CHD patients. RER overshoot was seen during recovery from CPET in all patients. CHD patients also showed decreased aerobic capacity, reduced cardiorespiratory efficiency, and a lower RER overshoot than controls. Patients with repaired transposition of the great arteries and tetralogy of Fallot, as well as those who underwent Fontan operation, had lower RER magnitude when compared with the controls and subjects who underwent repair of coarctation of the aorta. Based on the results of this study, the authors recommend that the RER recovery overshoot analysis should be an integral part of all CPET studies, and such an evaluation may help in the risk stratification of subjects with CHD.

In the second paper, Sganga and her colleagues from Harvard Medical School, Boston, MA, and Stanford University School of Medicine, Palo Alto, CA, USA review quality improvements in a pediatric echocardiography laboratory [3]. The authors assert that transthoracic echocardiography (TTE) is an important method in the diagnosis and management of CHD. The authors also state that TTE studies are challenging to perform and interpret because of the varying degrees of children’s cooperation, complexity of cardiac anatomy, and level of expertise of the interpreter. Consequently, errors in diagnosis are likely to take place. The authors devised a collaborative and stepwise quality improvement (QI) procedure to deal with errors in diagnosis in their echo lab. The authors performed a retrospective review of 100 consecutive cardiac surgery patients of less than five years old, seen from July 2020 to January 2021, to detect errors in TTE diagnosis. They detected eighteen (18%) errors in the diagnosis. Seventy eight percent (14/18) of these had minimal impact, and thirteen of the fourteen were likely to be preventable. They showed these results to their sonographers and echo interpreting faculty and invited their input on how to prevent and manage errors in diagnosis. The sonographers and faculty chose QI processes from among seven areas for perfection. The authors’ goal was defined as a 10% reduction in errors in diagnosis. Following the implementation of QI processes, errors occurred seven times in 70 subsequent studies; this represented a 44% decrease in error rate. The authors conclude that, by using a stepwise and team-based approach, QI process in the echo lab is feasible, and such an approach may serve as a model for a cooperative QI process at other hospitals. 

In the third paper, Dr. Pop from the University of Medicine Pharmacy Sciences and Technology of Tirgu Mures and the Tirgu Mures Emergency Institute for Cardiovascular Diseases and Heart Transplant, Tirgu Mures, Romania, compares different contrast agents (Iomeprol 350, Ioversol 350, Iopromide 370, and Iodixanol 320) used for computed tomographic angiography (CTA) studies in infants referred for aortic arch evaluation [4]. Dr. Pop asserts that CTA studies in babies are difficult, secondary to variations in the types of the contrast used and the volume and rate of infusion of the contrast material. She performed a retrospective comparison of 4 different contrast materials in 48 consecutive CTA studies in babies less than one year of age. All CTA studies were undertaken with the same 64-slice scanner and used similar power-injection techniques. The results indicated that Iodixanol 320 achieved nearly 40% less enhancement compared to the other three agents in identifying aortic coarctation and aortic arch hypoplasia [4].

In the paper to follow, I review clinical aspects of mitral atresia with normal aortic root [5]. Mitral atresia with normal aortic root is an uncommon complex CHD and comprises less than 1% of all CHDs. In this defect, the mitral valve atresia is present; an atrial defect, most often a patent foramen ovale, offers egress of the blood from the left atrium; either a single ventricle or two ventricles with hypoplastic left ventricle exist; and the aortic valve and aortic root are normal. Clinical, X-ray, and electrocardiographic data are non-diagnostic; however, echo-Doppler imaging is helpful in describing both the anatomy and pathophysiology. Other imaging studies are rarely needed. Therapy comprises focusing on the pathophysiologic abnormality caused by the defect itself and accompanying cardiac abnormalities at the initial presentation, usually in the neonatal period. These patients ultimately need staged Fontan (total cavo-pulmonary connection). The discussion included all three stages of Fontan. Complications seen between the stages of Fontan operation and after accomplishing Fontan procedure were also reviewed. Monitoring efforts are warranted for the speedy detection of these complications and to ensure these complications are swiftly attended. It was concluded that mitral atresia with normal aortic root can easily be diagnosed by the currently available diagnostic techniques and the lesion can be successfully addressed with present treatment regimens. 

In the next paper, Dr. Singh from the Baylor College of Medicine/The Children’s Hospital of San Antonio, San Antonio, TX, USA, reviews “as low as reasonably achievable” (ALARA) in pediatric electrophysiology laboratory [6]. Dr. Singh emphasizes the occurrence of adverse radiation effects (cataracts, skin abnormalities, malignancies, birth defects, and spine and orthopedic injuries) associated with electrophysiologic studies and therapy, particularly in children, and advocates ALARA. He also stresses and supports the abolition of needless exposure patients, physicians and other laboratory personnel to radiation with the simultaneously delivery of acceptable imaging for diagnosis and when offering intervention. He concludes that the use of fluoro-less or near-zero fluoroscopy and 3D electro-anatomical mapping techniques is likely to achieve ALARA.

In the sixth paper, Dr. Divekar and associates from the University of Texas Southwestern Medical Center/Children’s Medical Center, Dallas, TX, USA, and the Cleveland Clinic Children’s Hospital, Cleveland, OH, USA, review transcatheter device therapy and how to integrate advanced imaging with invasive procedures in the management of CHD [7]. These authors assert that percutaneous device placement is currently being performed as a primary treatment option for several CHDs that have previously been addressed only with heart surgery. The authors also state that new imaging technology is being utilized in the diagnosis, planning, guidance during the procedure itself, and when monitoring following device placement. They conclude that advocacy for the patient’s well-being and working in concert with regulatory agencies is germane for the further development of catheter-based device implantation in subjects with CHD.

In the subsequent paper, Dr. Arar and his colleagues at the University of Texas Southwestern Medical Center/Children’s Medical Center, Dallas, TX, USA, review the role of cross-sectional imaging in pediatric interventional cardiac catheterization [8]. This paper has similar attributes to the previous paper [7] but encompasses all pediatric catheter interventional procedures. Both MRI and CT, along with 3D reconstruction, are crucial methodologies and invaluable tools in pre-procedural evaluation and follow-up assessment and are likely to improve the outcomes of these procedures.

In the next paper, Dr. Betancourt and her associates from The Children’s Hospital of San Antonio/Baylor College of Medicine, San Antonio, TX, USA, review the utility of a three-dimensional printed model in the biventricular repair of complex CHDs [9]. They report a case of heterotaxy syndrome in which a 3D-printed prototype allowed them to better comprehend the complex cardiac anatomy and helped in pre-surgical planning and the execution of a successful biventricular repair. They conclude that the 3D-printed model is likely to advance the comprehension of anatomic complexities and accomplish successful surgical outcomes in complex CHD.

In a subsequent paper, Kepple and his associates from Creighton University School of Medicine and University of Nebraska Medical Center, Omaha, NE, USA, review the impact of extubation time on feeding outcomes after neonatal cardiac surgery [10]. The authors’ study objective was to assess the influence of the timing of extubation on feeding outcomes in newborn infants following surgical therapy for CHD. In this retrospective investigation, the study subjects (seen between December 2014 and June 2020) were categorized into three groups: 1. extubated in the operating room, termed immediate group; 2. extubated in the intensive care unit (ICU) between 0- and 3-days post-procedure, named the early group; 3. extubated beyond 3 days post-surgery, called the delayed group. When the immediate and early groups were compared, there was no difference in time to earliest enteral feed (1.3 days (1.0–3.4) vs. 2.3 days (1.1–3.3), *p* = 0.27). In addition, no difference in time to first oral feed (2.0 days (1.1–4.5) vs. 3.1 days (1.8–4.4), *p* = 0.34) and time to goal feed (6.0 days (3.2–8.3) vs. 6.9 days (5.0–9.0), *p* = 0.15) was detected. Furthermore, no variance in establishing all oral feeds in one year: 88% vs. 98%, *p* = 0.16 was identified. However, the delayed group performed substantially worse on all the above parameters. The authors conclude that immediate and early groups demonstrated no variations in feeding outcomes or length of stay in their investigation, while the delayed group performed worse on all measures. Consequently, they recommend that caregivers focus on extubating the babies within 3 days following surgery to enhance feeding outcomes while reducing hospitalization duration.

In the final paper of this series, Dr. Sharma and associates from The Children’s Hospital of San Antonio/Baylor College of Medicine, San Antonio, TX, USA, and the Cohen Children’s Medical Center, New York, NY, USA, discuss advances in the prenatal management of fetal cardiac disease [11]. They state that, despite the advances in fetal ultrasound studies that have improved the prenatal treatment of the heart, substantial issues remain regarding how the subjects are selected and the applicability of the available therapies. They review both pharmacological and percutaneous interventions in the fetus and suggest that these treatment modalities have improved remarkably over the years. However, they recommend that multi-institutional collaborative initiatives develop standardized methods for intra-uterine drug therapy and transcatheter interventions and create acceptable guidelines for fetal therapies to attain ideal results.

The papers included in this Special Issue, while not addressing all the advances that occurred in the last 50 years [1], have included a wide variety of diagnostic and therapeutic methods. The authors of these papers and I hope that the reviewed material is useful to the reader and help them to provide better care for their patients.

## Data Availability

Not applicable.
